# A multicenter study on clinico-epidemiological profile of phenylketonuria in Egyptian children

**DOI:** 10.1038/s41598-025-28353-3

**Published:** 2025-11-29

**Authors:** Sohier Yahia, Abdel-Hady El-Gilany, Rofaida M. Magdy, Gihan M. Bebars, Yossra S. Fadle, Heba Dawoud, Nesreen Safwat ELfeil, Mohamed M. Moussa, Rawan Assy, Abdelrahim A. Sadek, Zahraa Abdelmoneim

**Affiliations:** 1 Present Address: Department of Pediatrics, Faculty of Medicine, Mansoura University, Mansoura, Egypt; 2 Genetics and Metabolic Unit, Mansoura University Children’s Hospital, Faculty of Medicine, Mansoura University, Mansoura, Egypt; 3 Department of Public Health, Faculty of Medicine, Mansoura University, Mansoura, Egypt; 4 Metabolic and Genetic Unit, Department of Pediatrics, Faculty of Medicine, Sohag University, Sohag, Egypt; 5 Department of Pediatrics, Faculty of Medicine, Minia University, Minia, Egypt; 6 Department of Pediatrics, Clinical Genetics Unit, Faculty of Medicine, Tanta University, Tanta, Egypt; 7 Intern Doctor, Faculty of Medicine, Mansoura University, Mansoura, Egypt; 8 Oral Medicine, Periodontology, Oral Diagnosis and Oral Radiology Department, Faculty of Dentistry, Alexandria University, Alexandria, Egypt; 9 Neuropsychiatry Unit, Department of Pediatrics, Faculty of Medicine, Sohag University, Sohag, Egypt

**Keywords:** Phenylketonuria, Newborn screening, Neurodevelopmental complications, Epidemiology, BH4 deficiency, Multicenter study, Diseases, Health care, Medical research

## Abstract

Phenylketonuria is the most common heritable metabolic disorder. Early detection through newborn screening and proper nutritional management are essential for preventing neurodevelopmental complications. This study aims to describe the epidemiological profile of PKU in Egypt, assess the impact of early diagnosis, and examine the relationship between dietary adherence and comorbidities, including developmental and growth impairment. This is a multicenter retrospective cross-sectional study conducted in four university hospitals in Egypt between January 2024 and January 2025. A total of 365 patients with PKU aged 0–18 years were included. Data on demographics, phenotype classification, complications, and diet adherence were collected. We found that the most common PKU phenotype was classic PKU (36.3%). Early diagnosis through NBS was reported in 67.7%, and dietary adherence in 79.5%. Developmental delay was significantly lower in early-diagnosed children (3.2%) than in late-diagnosed children (100%). BH4 deficiency (1.6%) was associated with developmental delay and epilepsy despite early diagnosis. Diet adherence was linked to lower phenylalanine levels and fewer complications. Neurodevelopmental problems in PKU were decreased by the national NBS program. Better results depend on early diagnosis, diet adherence, and awareness of BH4 deficiency. Diet non-adherence not only worsens neurodevelopmental outcomes but also negatively affects growth parameters in these children.

## Introduction

Phenylketonuria (PKU) (OMIM 261600) is an autosomal recessive condition due to deficient activity of phenylalanine hydroxylase (PAH). This enzyme converts phenylalanine to tyrosine in the presence of tetrahydrobiopterin (BH4) cofactor^1^. PKU is the most common inherited metabolic disorder^2^.

Delayed diagnosis or neglecting treatment results in the elevation of phenylalanine (Phe) level in the bloodstream and brain. This causes mental retardation, developmental delay, psychological and behavioral disorders, epilepsy, eczema, and blond skin and hair.

Therefore, early diagnosis and treatment are crucial to prevent these complications of PKU^3^.

The national newborn screening (NBS) program targeting PKU achieved a brilliant success in the early diagnosis and treatment of PKU children. All newborns are screened for PKU by pricking their heels and collecting a blood sample on a filter paper card. This blood collection is usually done within 24 to 72 h or 3 to 7 days following birth. According to European guidelines for PKU diagnosis and treatment, NBS should be done for all newborns by taking blood samples (ideally between 24 and 72 h after birth) to ensure a timely start of treatment^4^.

The blood samples are subsequently sent to a public health laboratory for a thorough biochemical analysis. Individuals can have normal, healthy growth when early identification during the newborn period is combined with effective therapy to maintain metabolic balance. In 1960, the USA launched the first NBS program for PKU, and throughout the following years, it was adopted in many other countries^5^.

The first PKU screening initiative in Egypt was established in partnership with the Ministry of Health (MOH) in November 2015.

The primary treatment for PKU has historically been a phenylalanine-restricted diet. Current treatment techniques for this disorder emphasize strict metabolic regulation with a specialized low-phenylalanine diet that includes medical foods. Minimal amounts of Phe derived from breast milk or commercial infant formula are often deemed enough for babies^6^.

The clinical profile of PKU varies by ethnic group and geographic region. The total frequency in Europe is 1 in 10,000 infants, in Ireland 1 in 4500^7^, and in Turkey 1 in 2600^8^. No updated large-scale studies about PKU epidemiology in Egypt.

This work aims to describe the clinical-epidemiological profile of children with PKU, emphasize the importance of early diagnosis, and outline the role of NBS. It also highlights the significance of detecting dietary challenges and complications caused by either excess or deficiency of phenylalanine intake. Additionally, the study underscores the need for early suspicion of BH4 deficiency cases, as it is not included in the national neonatal screening program for healthy newborns but is a part of the NBS program targeting 19 genetic, metabolic, endocrine, and hematological diseases in sick newborns admitted to the nursery.

## Methods

This is a retrospective observational study done during the period from 1/2024 to 1/2025.

Target population: all infants and children (from birth to 18 years) diagnosed with PKU and followed up at Mansoura, Tanta, Mina, and Sohag University Hospitals.

Cases who lost follow-up, refused to share in the research, or had any other metabolic or genetic diseases were excluded.

### Sample size

All children with PKU aged 0–18 years who were monitored at the university hospitals of Mansoura, Tanta, Minia, and Sohag were included in the study. According to data from the local hospital registries, a total of 365 cases were identified and included.

Data were abstracted from patients’ records, including demographic data, including age, sex, and residency, family history, and consanguinity.

The age of diagnosis was either early through the NBS program and early treatment in the newborn period, or late, which refers to infants and children diagnosed with PKU after more than one month of age^9^.

The newborn screening used Whatman 903 filter paper to detect and dry blood from a heel stick for screening. The sampling period was between the third and seventh days of life. Dried blood spots were preprocessed according to the NeoBaseTM non-derivatized MS/MS kit instructions before being analyzed with the TQD MS/MS system and the NeoBaseTM non-derivatized system. The analyses include Phe, tyrosine, and the Phe: Tyrosine ratio. Positive cases underwent plasma amino acid analysis using chromatography to detect Phe levels^10^.

Our cases were classified according to the initial serum phe level into: classic PKU (serum phe plasma concentrations in an untreated, newly diagnosed newborn exceed 20 mg/dL (1200 micromol/L), moderate PKU (phe concentrations 900 to 1200 micromol/L) and mild PKU (phenylalanine concentrations 600 to 900 micromol/L), mild hyperphenylalaninemia (HPA, phenylalanine concentrations 360 to 600 micromol/L), benign mild HPA which doesn’t require therapy (phenylalanine concentrations 120 to 360 micromol/L)^11^,. The mean of their serum Phe level follow-up in the last three months was recorded. Treatment is indicated only in cases where with phenylalanine level is > 360umol/l^4^.

BH4 deficiency cases were suspected with their neurological manifestations, developmental delay, and or epilepsy despite diet adherence and were diagnosed by the BH4 loading test^12^.

Their weight and height were plotted on WHO growth charts. They were classified as average for age, which included those between the 5th and 95th percentiles, underweight or short stature for those below the 5th percentile, and overweight or tall stature for those above the 95th percentile for age and sex^13^.

We examined complications of PKU: neurodevelopmental delay and epilepsy by asking the parents and caregivers about their history of developmental milestones through the Denver Developmental Screening Test (DDST)^14^ and the presence of epilepsy.

The Denver Developmental Screening Test II (DDST-II) was used to do developmental screening on children ages 0–6. Developmental milestones for children older than six years were gathered from medical records and organized caregiver interviews.

In order to determine neurodevelopmental status in older children than six years, parents were also questioned about their children’s academic and school performance, a direct clinical neurological examination was performed, and school records were examined for evidence of cognitive and adaptive functioning. We recognize that not all older children had access to formal standardized psychometric testing, such as IQ tests, and we describe this as a limitation.

Furthermore, according to European guidelines for PKU diagnosis and treatment, clinical assessment of nutritional status is conducted at every outpatient visit, including anthropometric measurements and evaluation of clinical features of micronutrient and Phe deficiency—especially anorexia, listlessness, alopecia, and perineal rash. The frequency of these visits varies based on the patient’s age: for ages 0–1 years, every 2 months; for ages over 1 year, twice per year, provided there is good clinical and metabolic control^4^.

A dental assessment by a dentist was done to detect dental caries incidence in PKU children.

Dietary management for PKU includes phenylalanine-free formula (PKU1 for age less than 1 year, PKU2 for age 1 to 8 years, and PKU3 for more than 8years), a phenylalanine-restricted diet, and an amino acid-based medical food devoid of phenylalanine as a source of protein. The phenylalanine requirement is supplied from a low-protein diet. We give these amounts according to the calculated protein, phenylalanine, and tyrosine requirements according to age^15^. Diet adherence is determined by whether these infants and children follow these calculations or not.

This study was performed in line with the principles of the Declaration of Helsinki. The study protocol was approved by the Ethics Committee of Mansoura Faculty of Medicine-Institutional Research Board (Code number: R.25.01.3008), Sohag University: (Soh-Med-25-3—3PD), Tanta University: (approval code: 36264PR1139/3/25), and Minia University: (approval code: 1631).

### Statistical analysis

Data were analyzed using SPSS version 27 (IBM Corp., Released 2020. IBM SPSS Statistics for Windows, Version 27.0. Armonk, NY: IBM Corp). Categorical data were presented as numbers and percentages. The chi-square test or Fisher’s exact test was applied to investigate the association between categorical variables as appropriate. For continuous data, they were tested for normality by the Shapiro–Wilk test. For non-normally distributed continuous data, they were expressed in the median and interquartile range (25- 75th percentiles), and the Mann–Whitney U and Kruskal-Wallis tests were used for comparison between the studied groups. For normally distributed continuous data, were expressed as means ± SD, and the Independent t-test was used for comparison between the studied groups. Crude and adjusted risk ratios (prevalence ratios) and their 95% confidence intervals were calculated as risk measures. A p-value of < 0.05 was considered statistically significant.

##  Results

We studied the characteristics of 365 PKU patients; 186 (51%) were males and 179 (49%) were females. Positive family history was reported in 101 (27.7%) cases, and positive parental consanguinity is present in 76.2% [Table (1)].

The most common PKU phenotype reported in our study is classic PKU (36.3%). Most cases were diagnosed early through NBS (247 cases, 67.7%). Other clinical complications are reported in Table 1.

**Table 1:** Socio-demographic data and clinical characteristics of PKU patients.

	Number	Percentage
**Age in years** **Median (min-max)**	6 (0.25- 17)	
**Sex**		
**Male**	186	51%
**Female**	179	49%
**Residency**		
**Urban**	130	35.5%
**rural**	235	64.5%
**University**		
Mansoura	59	16.2%
Minia	60	16.4%
Sohag	173	47.4%
Tanta	73	20.2%
**Positive family history**	101	27.7%
**Positive consanguinity**	278	76.2%
**PKU phenotypes**		
Mild hyperphenylalaninemia	44	12.1%
Mild PKU	95	26%
Moderate PKU	88	24.1%
Classic PKU	132	36.3%
BH4 deficiency	6	1.6%
**Time of diagnosis**		
Early	247	67.7%
Late	118	32.3%
**Diet adherence**		
Adherent	290	79.5%
Non-adherent	75	20.5%
**Developmental delay**	126	34.5%
**Epilepsy**	20	5.5%
**Dental caries**	96	26.3%
**Protein malnutrition**	36	9.9%
**Weight**		
Below normal	32	8.8%
Average for age	284	77.8%
Below normal	49	13.4%
**Height**		
Below normal	49	13.4%
Average for age	271	74.2%
Below normal	45	12.3%

PKU = phenylketonuria, BH4 = tetrahydrobiopterin.

Our PKU patients were categorized into two groups: early diagnosed (*N* = 247) and late diagnosed (*N* = 118). There was a statistically significant difference between the two groups regarding age, developmental delay, and epilepsy, p-value < 0.001. Also, characterization of early diagnosed PKU cases presented by developmental delay is presented in Table 2.

**Table 2:** Comparison between early and late diagnosed PKU.

			Early *N* = 247		Late *N* = 118			*P* value	
**Age** **Median (IQR)**			5 (3–7)		10.8 (8–13.5.5)			< 0.001*	
**Developmental delay**			8		118			< 0.001**	
**Epilepsy**			5		15			< 0.001**	
**Diet adherence**			201		89			0.213**	
**Follow-up Phenylalanine level (umol/l)** **Median (IQR)**			275 (189–400)		325 (200–527)			0.120*	
developmental delay in early diagnosed PKU									

mild hyperphenylalaninemia	mild PKU	moderate PKU		classic PKU		BH4 deficiency	total		*P* value**
0	0	0		3		5	8		**< 0.001**

*Mann-Whitney U test, **Chi-square test.

A statistically significant p-value is bold.

Median follow-up phenylalanine level in early diagnosed PKU patients with normal development was 270 umol/l, but it was 505 umol/dl in those with delayed development. A statistically significant difference in the follow-up phenylalanine levels was reported between normally developed and development-delayed PKU children who were diagnosed early, with a p-value of 0.046.

Table 3 shows a comparison between different PKU phenotypes. There is a significant difference between follow-up phenylalanine levels and developmental delay between them, being highest in classic PKU.

**Table 3:** Comparison between PKU phenotypes.

	Mild hyperphenylalaninemia (*N* = 44)	Mild PKU (*N* = 95)	Moderate PKU (*N* = 88)	Classic PKU (*N* = 132)	*P* value
**Developmental delay**	15.9%	27.4%	29.5%	46.2%	< 0.001*
**Epilepsy**	2.3%	7.4%	4.5%	3%	0.389*
**FU phenylalanine** **(umol/l)** Median (IQR)	185 (150–252)	264 (167–400)	290 (222.8–450)	355 (210.5- 575.8)	< 0.001**

*Chi-square test, **Kruskal-Wallis test, statistically significant p-value is bold.

Table 4 shows the clinical characteristics of BH4 deficiency. BH4 deficiency was reported in 6 cases; five (83.3%) were diagnosed in the neonatal period. All cases had developmental delay, and epilepsy was reported in four (66.7%) cases. The Phenylalanine median level was 361.5 umol/l in BH4 deficiency cases.

**Table 4:** Clinical characteristics of BH4 deficiency.

	Number = 6	Percentage
**Time of diagnosis**	Early = 5	83.3%
	Late = 1	16.7%
**Developmental delay**	6	100%
**Epilepsy**	4	66.7%
**FU Phenylalanine level (umol/l)** **Median (IQR)**	361.5 (183.75–456.5)	

Table 5 shows a statistically significant difference between diet-adherent and non-adherent PKU children regarding follow-up phenylalanine level, developmental delay, dental caries, residency, underweight, and short stature.

**Table 5:** Clinical effect of diet adherence.

	Diet adherence *N* = 290	Non-Diet adherence *N* = 75	*P* value
**Follow-up phenylalanine (umol/l)** Median (IQR)	260 (180–351)	700 (589–800)	< 0.001*
**Developmental delay** N (%)	90 (31%)	36 (48%)	0.006**
**Dental caries** N (%)	60 (20.7%)	36 (48%)	< 0.001**
**Urban residency = 130** N (%) **Rural residency = 235** N (%)	111 (85.4%) 179 (76.2%)	19 (14.6%) 56 (23.8%)	0.037**
**Underweight** N (%)	25 (8.6%)	7 (9.3%)	0.009**
**Short stature** N (%)	38 (13%)	11 (14.7%)	0.022**

*Mann-Whitney U test, **Chi-square test, statistically significant p-value is bold.

Table 6 shows Factors associated with and significant independent predictors of developmental delay.

Table 7 shows a significant correlation between malnutrition, underweight, short stature, and dental caries.

**Table 6:** Factors associated with and significant independent predictors of developmental delay.

	Total *N* (column %)	Bivariate analysis		
		developmental delay *N* (row%)	*P*	CRR (95%)
Overall	365(100%)	126(34.5%)		
Age: <5 5-<10 10 & more	105(28.8%) 176(48.2%) 84(23.0%)	9(8.6%) 47(26.7%) 70(83.3%)	**≤ 0.001** **≤ 0.001**	r 0.1(0.05–0.19) 0.32(0.25–0.42)
Phe initial: 350–600 > 600–1200 > 1200	53(14.5%) 181(49.6%) 131(35.9%)	8(15.1%) 57(31.5%) 61(46.6%)	**≤ 0.001** **≤ 0.001**	0.32(0.17–0.63) 0.68(0.51–0.89) r
PKU type: Mild hyper-Phe Mild PKU Moderate PKU Classic PKU BH4 deficiency	44(12.1%) 95(26.0%) 88(24.1%) 132(36.2%) 6(1.6%)	7(15.9%) 26(27.4%) 26(29.5%) 61(46.2%) 6(100%)	0.14 0.09 **0.001** **≤ 0.001**	r 1.72(0.81–3.66) 1.86(0.88–3.94) 2.91(1.44–5.87) 6.29(3.19–12.4)
Early diagnosis: No Yes	118(32.3%) 247(67.7%)	118(100%) 8(3.2%)	**≤ 0.001**	r 0.32(0.02–0.06)
Adherence: No Yes	75(32.3%) 247(67.7%)	36(48.0%) 90(31.0%)	0.006	r 0.65(0.48–0.87)
Controlled PKU: No Yes	121(33.2%) 244(66.8%)	56(46.3%) 70(28.7%)	**≤ 0.001**	r 0.62(0.47–0.82)

CRR = Crude Risk ratio; ARR = Adjusted risk ratio, r = reference category, statistically significant p-value is bold.

**Table 7:** Correlation between protein malnutrition, dental caries, weight, and height in children with PKU.

	Protein malnutrition *N* = 36	No protein malnutrition *N* = 329	*P* value
**Underweight** N (%)	20 (55.6%)	12 (3.6%)	< 0.001*
**Short stature** N (%)	16 (44.4%)	33 (10%)	< 0.001*
**Dental Caries** N (%)	17 (47.2%)	79 (24%)	0.003*

*Chi-square test, statistically significant p-value is bold.

##  Discussion

PKU is the most common inherited metabolic amino acid disease. After the introduction of PKU screening in the national NBS program in Egypt, many hidden aspects of this prevalent disease became clear, leading to a fascinating area for research.

This multicenter study in Egypt included 365 PKU infants and children who were followed up in four university hospitals: Mansoura, Minia, Sohag, and Tanta, describing the clinico-epidemiological characteristics of these children, the effect of the introduction of the NBS program, available dietary management, and its challenges, and factors associated with developmental delay and growth impairment in these children.

There is no sex selection in affected infants and children, as this metabolic disease follows autosomal inheritance. 64.5% are from rural areas where consanguineous marriage is common.

Considering its autosomal recessive inheritance, consanguinity among carrier couples is viewed as the primary risk factor for PKU^16^. Additionally, variations in PKU prevalence across different communities may be linked to the frequency of consanguineous marriages in those regions^17^. Positive parental consanguinity was identified in most of our children. A positive family history is noted in 27.7% of cases, which includes features of autosomal recessive inherited diseases, where the recurrence of disease in the next sibling is 25%^18^.

As regards PKU phenotypes, the most common is classic PKU, followed by mild PKU, moderate PKU, and mild hyperphenylalaninemia, while BH4 is the least common type, occurring only in 6 cases (1.6%).

It copes with a study done by Hillert et al. (2020), who compared genotypes and metabolic phenotypes from 16,092 PKU cases in 51 countries across 17 world regions. They found that classic PKU is the most common (61.7%), followed by mild PKU (mild and moderate PKU categorized as mild PKU) at 22%, and mild hyperphenylalaninemia at 16%^19^.

Of our PKU children, 67.7% were diagnosed by screening through the national NBS program, while 32.3% were diagnosed late after the neonatal period by PKU manifestations. Late diagnosis can be explained by either older individuals before starting the national NBS program for PKU or by missing NBS at 3 to 7 days, either due to NICU admission or other issues. Other studies reported different percentages of late PKU diagnosis as Valle et al., 2019 (3.75%), Queiroz & Pondé, 2015 (50%), Figueira, 2018(43, 6%)^20^.

All late-diagnosed PKU children experience developmental delay, and 12.7% of them developed epilepsy, in contrast to only 2% of those diagnosed early. Gonzalez et al. (2011) reported that 46.3% of late-diagnosed PKU patients had intellectual disabilities, 28.5% were borderline, and 25% had normal IQ^21^.

It is important to consider that the severe cognitive impairments seen in untreated PKU can be partially reversed with dietary treatment in many individuals^22^.

The median age of lately diagnosed PKU is higher than those diagnosed early, as lately diagnosed PKU cases are before the introduction of the national NBS program. No significant differences were found in diet adherence and median phenylalanine follow-up between early and late diagnosed PKU children.

This demonstrates the importance of the national NBS program and how early diagnosis and treatment prevent developmental delay and epilepsy^23^.

Although neonatal screening and diagnosis prevent developmental delay in PKU children, eight cases of early diagnosis have developmental delay. Five cases of them are BH4-deficient.

By comparing the median follow-up phenylalanine levels between children with normal development and those with delayed development who were diagnosed early, it is observed that children with delayed development have significantly higher levels. This is because hyperpeylalaninemia causes cerebral toxicity by many mechanisms; reduction of protein and neurotransmitter synthesis due to competitive inhibition of a blood-brain-barrier (BBB) transporter of large neutral amino acids (LNAA), including tyrosine and tryptophan. Also, reduced cholesterol synthesis and myelin production, along with direct myelin toxicity, inhibition of tyrosine and tryptophan hydroxylase, complex reduction of glutamatergic synaptic transmission, inhibition of pyruvate kinase, and oxidative stress^4,24^.

As regards PKU phenotypes, the follow-up phenylalanine level and consequently developmental delay varied significantly among PKU phenotypes, with classic PKU showing higher levels than moderate, mild PKU, and mild HPA. This indicates that a higher initial Phe level leads to a higher follow-up Phe level. It can predict more challenging Phe level management, as well as additional problems and mental impairments in patients with elevated starting levels. This illustrates the harmful effects of long-term elevated Phe levels.

Discussing BH4 deficiency cases as a specific entity, as BH4 deficiency has a different pathophysiological mechanism in affecting mental development. We identified 6 cases in our study; although 5 of the 6 cases were diagnosed early in NBS and followed a restricted diet, all cases have developmental delay, and 4 cases have epilepsy.

These spots light that the neurological manifestations of BH4 deficiency are not due to hyperphenylalaninemia alone (as the median phenylalanine is 361.5 umol/l, upper normal) but also due to neurotransmitter dysfunction, as BH4 is an essential cofactor for other enzymes as tyrosine hydroxylase, tryptophan hydroxylases type 1 and 2^25^.

This leads to a deficiency of the brain neurotransmitters dopamine, serotonin, and norepinephrine. Dopamine is most frequently linked to reward-based learning and behavior as well as the regulation of voluntary movement. Arousal is modulated by norepinephrine, while serotonin primarily influences higher-order cognitive processes and behavior^26^. So screening for BH4 deficiency is crucial to prevent its neurological manifestations. Diagnosis is by reduced red blood cell DHPR activity (from dried blood spots) and/or abnormal urinary, dried blood spot, or cerebrospinal pterins, followed by molecular analysis if available^27^.

The mainstay of treatment for PKU has centered on limiting natural protein in the diet while supplementing with medical foods in order to prevent neurologic injury while promoting growth. Medical food usually supplies 75% to 85% of the protein needs of patients with PKU^28,29^.

Phe tolerance varies among PKU patients depending on the amount of residual PAH activity, as well as other factors such as age, growth, and activity level. Most patients with classic PKU tolerate < 500 mg Phe per day (10 g natural protein), while patients with mild to moderate PKU may tolerate < 1000 mg Phe per day (20 g natural protein). Protein tolerance has been shown to change throughout the life cycle, and thus periodic Phe tolerance reassessments should be conducted in order to optimize protein utilization in the body^4^.

Compliance with phe-restricted diet is always challenging; in our study, diet adherence is reported in 79.5% of PKU patients. Cost challenge is treated well in our centers as special Phe-free formula and medical foods are allowed for free. But other challenges, including poor palatability of protein substitutes and social isolation, are still affecting PKU patients’ daily lives. Most PKU patients included in our study are less than 10 years old (77%), and as mentioned in Walter et al. (2002), compliance with treatment decreased from 70% in childhood to 20% after the age of 15^30^.

In our work, we demonstrated the clinical effects of diet adherence, indicating that PKU children who adhere to their diet have more controlled Phe levels and a lower frequency of PKU complications (developmental delay, dental caries, underweight, and short stature) compared to non-diet adherent children. This confirms that strict dietary management is essential not only for maintaining normal Phe levels but also for preventing long-term physical and cognitive complications.

Children with PKU living in urban areas show greater adherence to dietary guidelines, which can be linked to their cultural, socioeconomic, and educational backgrounds.

Among the 290 PKU children who were adherent to diet and well-controlled, 90 exhibited developmental delay (of these, 89 children were diagnosed late, and 1 child had BH₄–deficient PKU).

This study demonstrated that neurodevelopmental outcomes are influenced not only by dietary adherence and phenylalanine (Phe) levels but also by the time of diagnosis and phenotypes of phenylketonuria (PKU), as shown in Table 6.

Most of our PKU children have normal growth parameters. This is consistent with a study conducted among twenty-two Taiwanese PKU patients, which found no significant difference between PKU patients and healthy controls regarding growth parameters^31^.

This demonstrates that early diagnosis and good dietary management lead to optimal growth parameters.

On the other hand, Ilgaz et al. reported that even with advances in dietary treatment, “optimal” growth outcomes are not achieved in PKU. Impairment in linear growth remains an issue, especially during early childhood and adolescence, but growth impairment is less likely in children with mild HPA^32^.

Although most of our PKU children have normal growth parameters, protein malnutrition is detected in 9.9% of our patients and is associated with significant underweight and short stature. Protein malnutrition occurred in the form of alopecia, dermatitis, edema, and diarrhea. It is caused by over-protein restriction due to fear of neurological manifestations of PKU, an unpalatable medical formula, and the high cost of protein substitutes. Also in PKU diet management, the amount of intact protein required to meet phenylalanine needs is often so limited that, without the use of a medical food, protein deficiency would develop^32^.

So, family education and the availability of medical formula are crucial.

Dietary management includes natural protein restriction because Phe is an essential amino acid present in most intact dietary proteins. Therefore, affected individuals should consume low-protein natural foods such as fruits, vegetables, sweets, and fats, along with industrially produced low-protein foods like bread and pasta, so they complete their caloric intake from a sugar- and carbohydrate-rich diet^33^.

Additionally, unpleasant medical formulas are often taken with sugary drinks to enhance the taste. These factors contribute to the increased vulnerability of children with PKU to dental caries.

This copes with a study done among 109 PKU patients, where they were found to be more vulnerable to dental caries when compared to healthy controls^34^.

Many factors can contribute to dental caries, including socioeconomic status, educational level, and fluoride intake. Therefore, it is crucial to identify the risk factors associated with caries and compare them with age-matched healthy controls. However, in our study, we only document the percentage of dental caries in PKU children and correlate it with diet adherence and protein malnutrition.

This study is considered the first in Egypt, including a multicenter study, to describe the clinico-epidemiological features of PKU infants and children, filling a significant gap in the national data.

A limitation of this study is its cross-sectional design, which lacks comparison between PKU and healthy infants and children regarding growth parameters, nutritional status, and dental caries. Further studies are needed on this issue.

## Conclusion

Phenylketonuria (PKU) is a common metabolic disorder in Egypt, presenting a significant issue as a treatable cause of intellectual disability and developmental delays. The introduction of the national newborn screening (NBS) program in Egypt in 2015 represented a crucial step toward diagnosing more cases, raising awareness of tetrahydrobiopterin (BH4) deficiency, ensuring the availability of medical formulas, understanding treatment challenges, and addressing the issues associated with over-protein restriction.

Indeed, neurodevelopment in PKU patients is influenced by multiple interrelated factors. While dietary restriction of phenylalanine remains the cornerstone of management, both dietary non-compliance and delayed diagnosis significantly lead to elevated phenylalanine levels and, consequently, neurotoxicity and neurodevelopmental delay. Furthermore, nutrient deficiencies secondary to over-protein restriction can further impair normal growth and development in these children. Our findings support this multifactorial influence, showing that even among diet-adherent patients, those diagnosed late or with specific PKU phenotypes (e.g., BH₄ deficiency) exhibited developmental delays.

Thus, we recommend improving public and medical awareness of PKU and its dietary management, supporting dietary adherence by ensuring the availability of medical formulas and protein substitutes, advising families on protein substitutes while making them appealing, and regular nutritional assessment. Also, early dental assessment is important. Furthermore, genetic counseling is an essential aspect of management, encompassing pre-marital counseling for all consanguineous marriages and providing support for couples with a family history of PKU.

## Data Availability

The datasets generated during and/or analyzed during the current study are available from the corresponding author on reasonable request.

## Abbreviations

BBB: Blood-brain barrier
BH4: Tetrahydrobiopterin
CNS: Central nervous system
DDST: Denver Developmental Screening Test
HPA: Hyperphenylalaninemia
LNAA: Large neutral amino acid
MOH: Ministry of Health
MS/MS: Tandem spectrometry
NBS: Newborn screening
PAH: Phenylalanine hydroxylase
Phe: Phenylalanine
PKU: Phenylketonuria
